# Math items about real-world content lower test-scores of students from families with low socioeconomic status

**DOI:** 10.1038/s41539-024-00228-8

**Published:** 2024-03-15

**Authors:** Marjolein Muskens, Willem E. Frankenhuis, Lex Borghans

**Affiliations:** 1 School of Business and Economics, Maastricht University & KBA Nijmegen, Nijmegen, Netherlands; 2 Evolutionary and Population Biology, Institute for Biodiversity and Ecosystem Dynamics, University of Amsterdam, Netherlands & Department of Psychology, Utrecht University, Netherlands & Max Planck Institute for the Study of Crime, Security and Law, Germany, Security and Law, Freiburg im Breisgau, Germany; 3 School of Business and Economics, Maastricht University, Maastricht, Netherlands

**Keywords:** Education, Psychology, Economics

## Abstract

In many countries, standardized math tests are important for achieving academic success. Here, we examine whether content of items, the story that explains a mathematical question, biases performance of low-SES students. In a large-scale cohort study of Trends in International Mathematics and Science Studies (TIMSS)—including data from 58 countries from students in grades 4 and 8 (*N* = 5501,165)—we examine whether item content that is more likely related to challenges for low-SES students (money, food, social relationships) improves their performance, compared with their average math performance. Results show that low-SES students scored lower on items with this specific content than expected based on an individual’s average performance. The effect sizes are substantial: on average, the chance to answer correctly is 18% lower. From a hidden talents approach, these results are unexpected. However, they align with other theoretical frameworks such as scarcity mindset, providing new insights for fair testing.

## Introduction

Despite longstanding policy efforts to reduce achievement gaps in education, socioeconomic status (SES) continues to be a strong predictor of academic performance^1–3^. In general, standardized math tests co-determine certification and admission to secondary and tertiary education. Performance on these tests is critical to academic achievement.

While standardized math tests are designed to measure math abilities, test items may carry unintended demands^4,5^. If personal characteristics, such as gender or SES, systematically impair or improve performance of people on particular items compared with other people *who have the same underlying ability*, test results are biased^6^. As indicated by the Standards for Educational and Psychological Testing, reducing test bias is crucial for reducing SES achievement gaps^7^. Fair testing thus requires minimalizing irrelevant features that selectively impede the performance of particular groups, such as low-SES students. For instance, it is well-established that language complexity in math items tends to lower the performance of low-SES students more than it does for high-SES students^8–10^. This knowledge has clear applied value: it enables designing fairer tests, which can reduce achievement gaps.

Here we ask: does the content of items on math tests, the story that explains a mathematical question, bias the performance of low-SES students? We address this question by examining whether particular types of content in math items is associated with better or worse performance among low-SES students compared with high-SES students (measured by the number of books in the home). Specifically, in a large-scale cohort study of Trends in International Mathematics and Science Studies (TIMSS)—which represents data from 58 different countries from students in grades 4 and 8 (*N* = 5501,165)—we examine the performance of low-SES students on items about content that may be particularly ecologically relevant for them (i.e., material that resembles real-world challenges and therefore has more meaning and consequence): money, food, and social relationships. We expected these types of content to improve the performance of low-SES students, relative to their individual’s average performance across all math items—as these contents are more likely to be associated with major challenges for low-SES students (e.g., lack of money and food, greater dependency on social networks, and higher levels of exposure to conflict). In the next section, we motivate this expectation and contrast it with deficit models, which emphasize the ways in which adverse experiences, which are on average more common in low-SES conditions, tend to undermine cognitive abilities.

It is well-established that students from low-SES backgrounds tend to score lower on math tests than high-SES students^1,2^; however, definitions and measures of SES vary between studies^11^. In this paper, we define SES as a person’s relative standing in society based on wealth and education^12,13^. Traditionally, SES is measured in youth through parental educational level, occupation, and income^14^. Other common measures include scales that capture people’s subjective assessments of their relative standing in society^15^, and measures of aspects of cultural capital, such as the number of books in the home^16^. In the current study, we used ‘the number of books in the home’ as an indicator of SES. This measure captures a key component of SES, namely the position related to the level of education and more specifically, cultural capital. This indicator is frequently used and recommended in cross-national educational research^17–20^, because it shows moderate correlations (in the range 0.3 to 0.4) with other key components of SES, such as access to financial resources and parental occupational prestige, in a wide range of countries^16,21^. However, this measure also has several limitations, discussed later (see Methods section).

It is crucial to distinguish between SES and the factors and processes explaining associations between SES and particular outcomes, such as academic performance. People in low-SES conditions have diverse experiences, both between and within societies. However, compared with people in high-SES conditions, they are more likely to be exposed to various forms of adversity, defined as negative experiences that pose a significant challenge to an individual’s goals and well-being^22–24^. These experiences may include having limited or unreliable access to material resources (e.g., money, food) needed to meet basic needs^25–32^ and higher levels of exposure to threat, such as family and neighborhood violence^24,26,33^.

Having acknowledged the complexity of SES and its correlates, it is clear that systematic and structural factors contribute to the relations between SES and educational performance^34^. These include low-SES students on average being exposed to higher levels of childhood adversity and having fewer learning opportunities in school that prepare them for achieving academic success^35^. In addition, poverty can directly impede cognitive functioning by imposing cognitive load that distracts attention and reduces effort^36^. While acknowledging such factors, deficits may not be the whole story. Specifically, deficit models lack a focus on the ways in which adaptive developmental processes may *shape*, rather than impair, cognitive abilities in contexts of adversity; that is, tailor abilities based on experiences for solving recurrent challenges faced in such contexts.

A recent synthesis of evidence from history, anthropology, and primatology shows that over human evolution, people have faced large variation in the extent of adaptive challenges such as threat (i.e., experiences involving the potential for harm imposed by other agents) and social, cognitive, and nutritional deprivation (i.e., low levels in the quantity and quality of social, cognitive, and nutritional inputs, respectively) across space and time^37–39^. These conditions likely favored a high degree of phenotypic plasticity, that is, the ability to tailor the brain, cognition, and behavior to different conditions, including adverse environments. For this reason, we should expect these forms of adversity to not only impair cognition, but also to shape it in ways that help people navigate meaningful challenges in their environments.

The hidden talents approach proposes that adaptive developmental processes might result in certain cognitive abilities being enhanced, rather than impaired, by adversity^40–43^. For instance, some studies show that children who have been exposed to high levels of violence are able to detect threats (e.g., angry facial expression) faster and more accurately than children exposed to lower levels of violence^44^. However, the evidence for most hidden talents is currently limited: there is some evidence for some abilities, but in most cases the evidence is mixed^42^. A general pattern found in several studies of hidden talents to date, however, is that the performance of people living in conditions of poverty or adversity, or both, benefits more from ecologically relevant materials than the performance of people from more favorable environments does^40–43^. For instance, in a study on executive functioning, youth exposed to higher levels of poverty and violence scored lower on an abstract working memory updating task (consistent with deficit models), but this performance gap nearly closed with ecologically relevant content (i.e., money, an angry face, and a school bus)^45^. Such equalization, and even enhanced performance, has also been observed in a study using only abstract contents (geometric shapes), when uncertainty was experimentally induced in people exposed to higher levels of unpredictability^46^. Other studies have found a similar pattern without an induction of uncertainty^47,48^. Together, such findings are striking compared with the existing literature in developmental science, which has nearly exclusively reported lower scores on cognitive tasks by people living in adverse conditions.

An exception to this tenet is a body of research from anthropology and cultural psychology showing that people living in low-SES conditions, who tend to have less exposure to formal education, are able to solve many complex cognitive challenges in real-world settings with ecologically relevant content, but struggle to solve equivalent challenges in educational settings^41,49–51^. For instance, this work has shown that children living in adverse environments may show mathematical abilities in real-world contexts that standardized tests do not capture. For instance, Schliemann and Carraher^52^ showed that Brazilian children living in poverty and adversity—many of whom were homeless—can solve math problems fast and accurately with concrete objects (e.g., fruits) while selling goods on the market, but performed substantially less well in a formal test setting using paper-and-pencil assessments with abstract contents (e.g., numbers). Banerjee and colleagues^53^ recently replicated this finding in India with youth living in poverty. In the United States, research with gifted youth shows that students from low‐SES backgrounds may have a preference for concreteness and practical applications in learning^54^.

Jointly, these studies suggest that children living in adverse conditions may develop cognitive abilities that are untapped by the school system, which can be exposed using ecologically relevant test settings and materials. Mapping these untapped abilities and their manifestations in different contexts is key for moving towards a well-rounded view of people who live with adversity, which incorporates performance deficits and strengths^42^. As we discuss later, such a well-rounded view has the potential to reduce stigma^40^, which in turn can have beneficial effects on low-SES students’ academic persistence, by supporting their motivation and beliefs about themselves^55^ and by promoting educators to better understand these youth’s strengths and potential for academic learning and performance^56^.

Based on the findings just reviewed, we hypothesized that low-SES students might perform better on math test items with ecologically relevant content compared with their average performance on all math items. To illustrate, the question to “divide 240 by 6” includes only mathematical content, whereas “distribute 240 euro among 6 friends” additionally includes content about money and social interaction. We selected three different types of content that we thought to be particularly ecologically relevant for low-SES students compared with high-SES students: money, food, and social interactions. We selected these types of content based on empirical literatures about the challenges associated with living in low-SES conditions (as discussed below). Moreover, these types of content are commonly used in items on standardized math tests.

First, people in low-SES conditions are more likely to experience limited or unreliable access to economic resources^26^, and lower levels of job stability, than people in high-SES conditions^27,28,31,32^. Second, due to their limited or unreliable access to economic resources, people in low-SES conditions are also more likely to experience food insecurity, limited or uncertain access to adequate food^25,30^. Although severe hunger is a typical consequence of disasters such as war, drought, or earthquakes, in all countries—including those that have few disasters and a relatively high standard of living—food insecurity is related to poverty^25,30^. About 736 million people worldwide live in poverty^57^. Third, due to limited or unreliable access to resources, people in low-SES conditions are more dependent on other people for their basic needs. Accordingly, cultural psychologists have argued that people in low-SES conditions are particularly attuned to other people. Specifically, people living in low-SES conditions may prioritize external, social factors in the environment over internal, individual factors^32^. However, other people are not only a source of support: people in low-SES conditions are also more likely to experience various forms of threat (e.g., family and neighborhood violence), which may further increase their attunement to social information. Thus, people in low-SES conditions may have a greater focus on social relationships, social networks, hierarchy, and the thoughts and intentions of other people^29,32,58^.

As noted, we expected ecologically relevant content to improve the test performance of low-SES students more than that of high-SES students. However, there are other perspectives that provide different, or even opposing, predictions. These perspectives did not initially guide our research, in part because we were less familiar with them; but they are just as relevant, and their predictions are better aligned with the findings of our study (discussed later). We now turn to these three perspectives.

First, from an attentional processes’ perspective, highly valuable content in the face of scarcity may distract students from their task, and narrow attention and cognitive control, which in turn might reduce their math performance^59–61^. From this perspective, valued resources such as money and food might affect attentional processes, potentially accompanied by rumination. This perspective aligns with findings of a recent study, which focused on the effects of monetary salience in mathematic exams on the performance of socioeconomically disadvantaged students^62^. This study demonstrates that low-SES students perform worse on items with money content using both TIMSS and other datasets. Moreover, this study provides evidence for spill-over effects. Leveraging the randomized ordering of questions in math tests, the monetary salience of items affects performance on subsequent items. These spill-over effects suggest that the content of an item can influence not only performance on the item itself, but also students’ ability to perform on subsequent items, which are not financially salient. Duquennois^62^ notes that such spill-over effects may be explained by a scarcity mindset—that is, poverty capturing attention and/or generating intrusive and distracting thoughts, reducing an individual’s cognitive resources^61,63^—with financial content in particular causing ‘attention capture’, which interferes with immediate and later performance.

Second, it is well established in educational science and practice that concrete everyday examples in math education can be challenging for both low- and high-SES students. In fact, many students have difficulty using their real-world knowledge when solving word problems in school^9,64^. Also, on average, students tend to be quite successful in solving simple world problems that can be solved using a single operation (i.e., addition, subtraction, multiplication, division). However, more complex word problems, which cannot be solved by a single routine application, create difficulties for most students^9^. Such difficulties may result from having to transfer from informal to formal skills and knowledge. When using everyday examples in word problems, students must go beyond the associations they have with the examples themselves (e.g., between cake and birthday parties), and draw analogies between the informal examples and the arithmetic algorithms learned in school (e.g., between cake and adding fractions)^65,66^. Research on the effects of concrete examples on performance suggests that the more salient an example is, such as toys or candy, the more difficult it is to go beyond the informal representation^67,68^. This perspective predicts that ecologically relevant content in math test decreases students’ performance.

Third, whereas attention capture effects and difficulties with transference from informal to formal knowledge may lead to a negative relationship between the use of ecologically relevant content and student performance on tests, there might also be affective mechanisms at play. Specifically, stereotype threat may play a role, if students belonging to a marginalized group perform less well when they receive cues that remind them of their stigmatized group identity^69–72^, such as items about money or food. Moreover, reminding people who belong to marginalized groups of their background-specific strengths—which may reduce stereotype threat—can increase their feelings of empowerment to succeed in school, engagement in their courses, sense of belonging, and academic persistence^55,73–75^.

In the current study, we examine whether low-SES students perform better or worse on math items about money, food, and social interaction compared with items about other types of content. We started the current study with the expectation that the use of ecologically relevant content would enhance low-SES students’ performance on math tests, based on predictions from the hidden talent approach. After conducting these pilot analyses based on 20 items with ecological relevant questions and 20 neutral questions of the TIMSS, as well as a replication of the ecological relevant questions using 1999 and 2003 TIMSS data, where we found the opposite of what we initially expected and preregistered, we explored a larger dataset of 161 items in the TIMSS to examine how robust and strong our findings results are, described below. The pilot results are included in the supplementary information.

In many countries, math tests are used in all stages of students’ educational pathways to tertiary education. Performance on math tests plays a crucial role in determining certification and admission to secondary and tertiary education. Therefore, we test our research questions using cross-national data. Specifically, we test at the student-level whether there is an interaction effect between students’ SES and content about money, food, and social interaction test items on math performance, controlling for potentially relevant features of items and students’ countries. In addition, we test on the item-level whether items with content about money, food, and social interaction show bias to the advantage of low-SES students relative to other types of content, controlling for features of items known to affect the math-performance of low-SES students or second language learners.

## Results

### Results on student-level

Our analyses included data from 58 countries including students in grades 4 (average age 9.5) and 8 (average age 13.5) (*N* = 5501,165) who completed math-tests from Trends in International Mathematics and Science Studies (TIMSS), wave 2007 and 2011. We identified items with ‘low-SES ecologically relevant content’ as items with mathematical problems involving 1) money), 2) food, or 3) social interaction (e.g., competition, working together). We define all remaining items as items with ‘low-SES neutral content’. These are items with mathematical problems involving 1) word problems with neutral content (e.g., buttons, frogs) or 2) mathematical notation (e.g., 5631 + 286 = …).

We conducted mixed logistic regressions analyses with performance on an item (1 = correct answer, 0 = incorrect answer) as a dependent variable, students SES-background (scale 1–5; 1 = low, 5 = high, dummy coded) with relevance category (1 = low-SES relevant, 0 = low-SES neutral) as an interaction term, and students SES-background (scale 1–5; 1 = low, 5 = high) and their individual test score as between subjects factors. In addition, we included relevance category ((1 = low-SES relevant, 0 = low-SES neutral) as a within subjects factor, and features of items (word problem, item type, context domain, cognitive domain – Knowing, Applying, and Reasoning–, total word count, number of different words, total number of characters, number of characters without spaces, average syllables per word, sentence count, average sentence length, academic words, quantitative language, spatial language, and country-dummies) as covariates. We conducted these analyses separately for grades 4 and 8. In separate regressions low-SES relevant content has been replaced by a dummy indicating questions about money, food, or social interactions, leading to four regressions for each grade. The estimates for low-SES relevant content are larger than 1, implying that for the highest SES-group (the reference category), these questions tend to be easier than the average question. Table 1 reports the estimate of the interaction of the dummy for the lowest SES-group and low-SES relevant content. The highest SES-group is the reference category. This estimate thus reveals how much lower the chance is of a correct answer when low-SES children answer low-SES relevant questions, conditional on the total score of the child and the difficulty of these questions, compared to high-SES children.

**Fig. 1:**
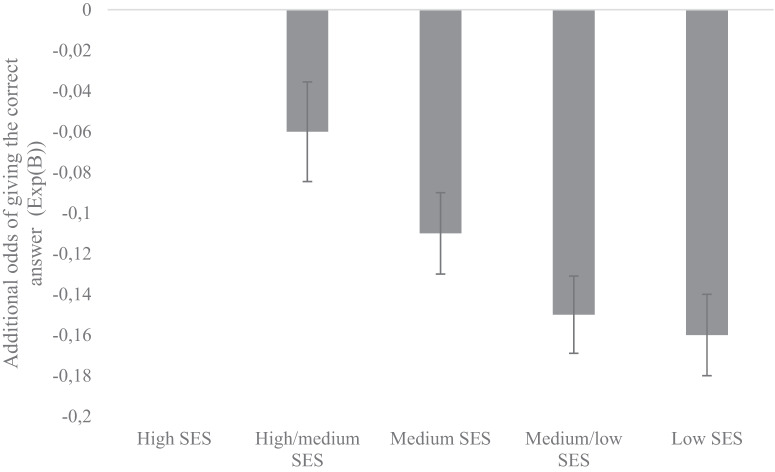
Interaction between SES-background and all low-SES ecologically relevant content in grade 8: the decrease of students’ odds to report the correct answer compared to high-SES students. In the model, SES (scale 1–5; 1 = low, 5 = high, dummy coded), relevance category ((1 = low-SES relevant, 0 = low-SES neutral), and the interaction SES x relevance category were included, controlling for students’ math ability, word problem, item type, context domain (3 dummy variables), cognitive domain (2 dummy variables), total word count, number of different words, total number of characters, number of characters without spaces, average syllables per word, sentence count, average sentence length, academic words, quantitative language, spatial language, and country (58 dummy variables) (grade 8, results from grades 4 and 8 show the same pattern). High SES is the reference category. Bars represent additional odds of giving the correct answer. Error bars represent one standard error of the mean.

In contrast to preregistered predictions, analyses show both in grade 4 and grade 8 (Table 1) a significant interaction between SES-background and low-SES ecologically relevant content (i.e., money, food, and social interaction), indicating that students from the lowest SES-background have a 16% (grade 8, Exp (B) = 0.84) and 18% (grade 4, Exp (B) = 0.82) lower chance of correctly responding to items with low-SES ecologically relevant content than students from the highest SES-background, given their average math performance (see also Fig. 1). In addition, we have conducted the same analyses but separately for low-SES ecologically relevant content money, food, and social interaction. Results for the interaction-terms are also shown in Table 1. These results indicate that the interaction we found depends specifically on content about money and social interaction in both grade 4 and 8, and on content about food in grade 4 (but not in grade 8).

**Table 1:** Results from logistic repeated measures analyses predicting change of giving the correct answer among students in grade 4 and grade 8 from different SES backgrounds by relevance of content of items, separate analyses for money, food, and social interaction

	Grade 4		Grade 8	
	Exp (B)	95% CI		95% CI
Low-SES relevant content (all) × SES	0.82*	[0.80, 0.85]	0.84*	[0.84, 0.88]
Money × SES	0.82*	[0.79, 0.86]	0.85*	[0.85, 0.90]
Food × SES	0.78*	[0.75, 0.82]	1.15	[1.04, 1.28]
Social interaction × SES	0.88*	[0.85, 0.92]	0.85*	[0.82, 0.87]
*N*	2779383		2721782	

*Note*. Interaction between lowest SES (1) and highest SES (5) and low-SES ecologically relevant content. In all models, SES (scale 1–5; 1 = low, 5 = high, dummy coded), relevance category ((1 = low-SES relevant, 0 = low-SES neutral), and the interaction SES x relevance category were included. The following covariates were included: students’ math ability, word problem, item type, context domain (3 dummy-variables), cognitive domain (2 dummy-variables), total word count, number of different words, total number of characters, number of characters without spaces, average syllables per word, sentence count, average sentence length, academic words, quantitative language, spatial language, and country (57 dummy-variables in Grade 4, 58 dummy-variables in Grade 8), CI = confidence interval, *p < 0.001.

Figure 1 provides, as an example, all the coefficient of the interactions between SES-categories and the dummy for SES-relevant content. Since high SES is the reference category this estimate equals 0. The estimate for low SES equals −0.16 which is equal to the exp(B) of the reported value in Table 1 for all low-SES relevant content in grade 4. The other SES-levels have estimates that vary gradually in between these two extremes.

### Results on item-level

To detect if ecologically relevant content biases test results on item-level, we conducted Differential Item Functioning (DIF) analyses for SES background. Items show DIF if students from different backgrounds with the same average score on a math test have a different probability of giving the correct response on a specific item.

As preregistered, we first conducted three studies with a small sample of randomly selected items. Results of these initial preregistered studies can be found in the supplementary materials.

Next, we analyzed with two complete tests from TIMSS 2007 and 2011 on item level (161 items) whether DIF to the disadvantage of low-SES students occurs statistically more in items with low-SES ecologically relevant content, controlling for other relevant features of items, such as linguistic complexity.

Table 2 shows descriptive statistics of the items. Table 3 shows differences between items with and without low-SES ecologically relevant content on several features. We compared items with low-SES ecologically relevant content with other items with neutral content (word-problems, and items with only mathematical notation). Table 3 shows that math items containing low-SES ecologically relevant content more often show significant DIF (Mantel-Haenszel (MH)) to the disadvantage of low-SES students than items containing word problems with other content and items with only mathematical notation. The MH can be interpreted as the probability for students with low SES to answer correctly, given their average math performance. When the value is below 1.00, the chance of giving a correct answer is lower than predicted based on their ability, if the value is higher than 1.00, the chance is higher than predicted based on their ability. The odds for low-SES students to respond correctly, in comparison to high-SES students and given their average math performance, is significantly lower (0.91) in math items with low-SES ecologically relevant content than in the other items (1.02 and 1.06) (see Table 3).

**Table 2:** Descriptive Statistics of 161 Math Items from TIMSS 2007 and 2011

	Proportion or mean	SE	Min	Max
Grade				
Grade 8	0.55	0.04	0	1
Grade 4	0.45	0.04	0	1
Item-type				
Multiple Choice	0.53	0.04	0	1
Open response	0.47	0.04	0	1
Context domain				
Algebra	0.11	0.02	0	1
Data and Chance	0.11	0.02	0	1
Data Display	0.06	0.02	0	1
Geometric Shapes and Measures	0.15	0.03	0	1
Geometry	0.14	0.03	0	1
Number	0.44	0.04	0	1
Cognitive domain				
Knowing	0.35	0.04	0	1
Applying	0.46	0.04	0	1
Reasoning	0.19	0.03	0	1
Type of problem				
Word problem	0.90	0.02	0	1
Mathematical notation	0.10	0.02	0	1
General language features				
Total word count	15.88	0.88	0	57
Number of different words	12.31	0.60	0	40
Total number of characters	153.61	8.60	7	579
Number of characters without spaces	83.14	4.82	0	347
Average Syllables per Word	1.47	0.02	0	2
Sentence count	3.66	0.17	1	12
Averages sentence length in words	9.69	0.34	0	26
Academic word use (AWL)	0.34	0.04	0	1
Math language features				
Quantitative language	0.55	0.04	0	1
Spatial language	0.45	0.04	0	1
Low-SES ecologically relevant content				
General (all low-SES relevant content)	0.32	0.04	0	1
Money and trading	0.13	0.03	0	1
Food	0.04	0.02	0	1
Social interaction	0.17	0.03	0	1
Differential Item functioning (DIF) to the disadvantage of low-SES				
MH	0.99	0.02	0.57	1.56

*N* = 161 items. *MH* Mantel-Haenszel. From all items, 32% contain low-SES relevant content (all content). From these items, 2% contains more than one type of low-SES relevant content.

**Table 3:** Differences between items with low-SES ecologically relevant content (money, food, and social interaction) and items with neutral content (word problems and items with only mathematical notation) on potentially relevant features and Differential Item Functioning (DIF)

	Low-SES relevant content	Neutral content	
		Word problems	Mathematical notation
Grade			
Grade 8	0.57%	0.50%	0.75%
Grade 4	0.43%	0.50%	0.25%
Item-type			
Multiple Choice	**0.33%**	0.57%**	0.94%**
Open response	**0.66%**	0.43%**	0.06%
Context domain			
Algebra	**0.04%**	0.09%	0.44%*
Data and Chance	0.22%	0.06%	0.00%
Data Display	**0.14%**	0.01%**	0.06%
Geometric Shapes and Measures	**0.04%**	0.21%**	0.13%
Geometry	**0.02%**	0.21%**	0.06%
Number	0.55%	0.41%	0.31%
Cognitive domain			
Knowing	0.24%	0.36%	0.69%*
Applying	0.45%	0.51%	0.19%*
Reasoning	**0.31%**	0.13%**	0.13%**
General language features			
Total word count	**20.71**	15.12**	4.94***
Number of different words	**15.61**	11.87**	4.38***
Total number of characters	**202.12**	144.48**	52.69***
Number of characters without spaces	**110.65**	78.63**	22.00***
Average Syllables per Word	**1.50**	1.50	1.25**
Sentence count	**4.45**	3.49*	2.19**
Averages sentence length in words	**10.86**	9.91	4.67***
Academic word use (AWL)	0.39	0.34	0.19
Math language features			
Quantitative language	**0.76%**	0.51%**	0.06%**
Spatial language	0.47%	0.47%	0.25%
Differential Item Functioning (DIF) to the disadvantage of low-SES			
MH	**0.91**	1.02***	1.06*

To examine the differences between items with low-SES relevant content and neutral content (word problem and mathematical notation), we conducted multivariate ANOVA with item type (low-SES relevant, word problem, mathematical notation) as predictor and the other characteristics of the items as dependent variables (two-sided). We conducted these analyses separately for low-SES relevant vs. word problem, and low-SES relevant vs. mathematical notation. So, all significance tests are compared to items with low-SES relevant content (first column). Bold numbers indicate a significant difference *P < 0.05, **P < 0.01, ***p < 0.001, *N* = 161 items.

To test whether these differences remain significant after controlling for other features of items that can affect low-SES students’ performance, we conducted linear regression analysis with DIF-odds (MH) as dependent variable, low-SES relevance as predictor (1= low-SES relevant content, 0 = neutral content (non-relevant word problems + mathematical notion)), and all relevant variables (bold) in Table 3 as covariates. Results show that for items with low-SES ecologically relevant content, the difference in the odds of reporting the correct answer between low-SES and high-SES students’ is larger, controlling for their overall performance on the test and controlling for other features of items (*b* = −0.09, t(160) = −2.55, *p* = 0.012, Cohen’s *d* = 0.70). Since the sample size of this analysis on item-level is 161, this effect size is meaningful. Cohen’s *d* indicates a medium effect size (0.70).

**Fig. 2:**
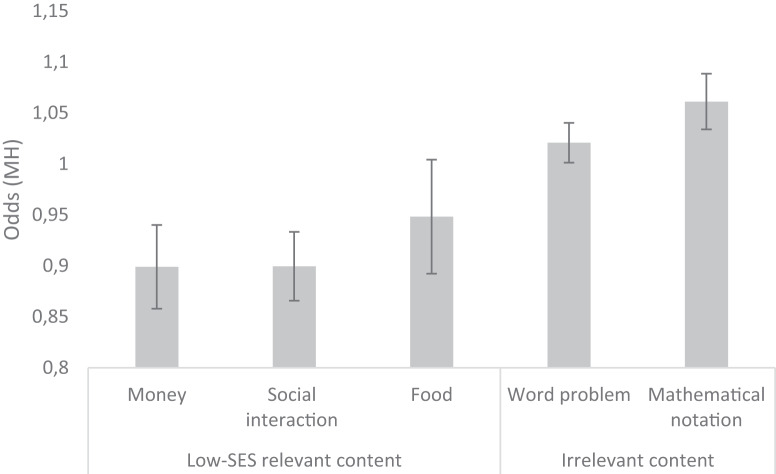
Odds resulting from DIF-analysis (Mantel-Haenszel procedure) on SES background and content of math items. Results show that items with low-SES ecologically relevant ‘money’ and ‘social interaction’ versus neutral content (word problems and mathematical notation), and items with ‘food’ versus mathematical notation, have a lower chance of being correctly answered by low-SES students, compared to high-SES students with the same math ability (161 items). Error bars represent one standard error of the mean. All analyses remain significant after controlling for multiple testing using a Bonferroni correction.

In addition, we analyzed DIF-odds separately for our relevance categories: money, food, and social interaction. Results suggest that especially items containing content that refers to money and social interaction are related significantly to a lower chance of correctly responding among low-SES students compared to high-SES students with the same math ability (Fig. 2). In addition, results suggest that items containing content that refers to food—compared with items with only mathematical notation—are related significantly to a lower chance of correctly responding among low-SES students compared to high-SES students with the same math ability. However, content related to food did not show a significant difference with word problems with neutral content.

## Discussion

Overall, our findings unexpectedly suggest that content in math test items related to money, food, and social interactions, hinders low-SES students’ performance. Compared to items with neutral content, low-SES students were less likely to correctly answer items with ‘ecologically relevant’ content than expected given their average math ability. The effects are substantial: students from low-SES backgrounds are on average 16% (grade 8) to 18% (grade 4) less likely to respond correctly when items contain this relevant content, given students’ average test score. These effects cannot be explained by linguistic complexity, nor by differences in content domain or cognitive domain (Knowing, Applying, and Reasoning) between items.

Our findings are unexpected from a contextual perspective, according to which students may learn and perform better when content and problems in standardized tests match with their practical knowledge and adaptive competences^40,43,51,76–79^. In formal school settings, low-SES students are more likely than high-SES students to experience a mismatch with the skills and knowledge they have learned in their home environments^40^. Therefore, it is important to examine whether problems involving content that is more ecologically relevant for low-SES students may promote their performance on math tests. We expected that test items involving content like money, food, and social interaction, which are relevant for low-SES students (more so than abstract content, such as numbers), would enhance their performance. However, we found the opposite effect: content relevant for low-SES students disadvantaged their math performance.

Our findings align with those of a recent study, which focused on the effects of monetary salience in mathematic exams on the performance of disadvantages students^62^. This study also finds that low-SES students perform worse on items with money content using both TIMSS and other datasets. Duquennois^62^ explains the results by a scarcity mindset with financial content that causes “attention capture,” which interferes with performance^61,63^. This ‘attention capture’ explanation may also be applied to our finding that low-SES students performed less well on items about social relationships and food, assuming that attention capture occurs more generally for content associated with negative thoughts and feelings (e.g., tense relationships, food scarcity). While we recognize that attentional processes likely play a critical role in our unexpected findings, applied educational science and fundamental cognitive psychology provide additional potential explanations and directions for future research, which we discuss below.

In educational science and practice, it is assumed that difficulties with word problems can result from difficulties with transference between informal and formal skills and knowledge. When using everyday examples in word problems, students must go beyond the associations they have with the examples themselves, and draw analogies between the informal examples and the arithmetic algorithms learned in school^65,66^. And, the more salient an example is—such as candy or toys—the more difficult it is to go beyond its informal representation^67,68^. The role of salience of content in solving word problems may be particularly important in explaining our unexpected findings, because content related to money, food, and social interaction content might be more salient for low-SES than for high-SES students.

Our initial expectation that ecologically relevant content would enhance, rather than hinder, low-SES students’ performance on math tests was informed by studies showing enhanced performance using ecologically relevant content among youth exposed to adversity^42,45^. A more recent study of executive functioning found that youth who had experienced relatively high levels of poverty and violence scored lower on an abstract working memory updating task than other youth. However, this achievement gap was nearly closed when using ecologically relevant content^45^. This study provides another instance of ecologically relevant content in tests promoting low-SES students’ performance. However, given the results of our current study, we may speculate that other processes caused by ecologically relevant content in math items can override or counteract the benefits of such content for performance on working memory^45,46,48^ and cognitive flexibility tasks^47^.

Research showing enhancement of low-SES students’ performance by ecologically relevant content has focused on components of executive function, such as attention shifting, inhibition, and working memory, which students develop in their home environments, prior to and without formal training in school^80^. In contrast, the TIMSS math tests used in the current study measure math skills and knowledge that does require formal schooling, alongside measuring executive function. We may speculate based on our unexpected findings that the use of ecologically relevant stimuli improves performance on tests that require skills that students also use in their home environments, but at the same time hinders their performance when a test requires to draw analogies between the informal examples and the arithmetic algorithms learned in school. In addition, because money, food, and social relations are likely to be highly salient for low-SES students, specifically this content may hinder their ability to go beyond the informal representation and use their math skills learned at school.

In finding out how and when the content of math items can hinder the solving of a math problem, we may distinguish between four phases of mathematical problem-solving: 1) understanding the problem, 2) devising a plan, 3) carrying out the plan, and 4) evaluation^81^. Given the mixed evidence on the effects of ecologically relevant content on low-SES students’ performance, future research should explore the possibility that ecological relevant content increases performance in one phase, while also hindering performance in another. For example, McNeil et al.^68^ found that among fourth- and sixth-grade student, more salient everyday examples can lead to better conceptual understanding of a mathematical problem (phase 1), which aligns with the contextual perspective on performance^40,43,51,76–78^. At the same time, this experimental study showed that salient everyday examples can lead to more arithmetic errors (phase 3), supporting the attention capture hypothesis^62^. This exploration related to the phases of mathematical problem solving is also important for practical applications, because the various explanations we see now lead to different possible interventions during test taking (e.g., increase attention to understanding the problem, or fostering greater conscientiousness when doing calculations).

Whereas attention capture effects and difficulties with transference from informal to formal knowledge provide possible explanations for our findings, stereotype threat—if students belonging to a marginalized group perform less well when they receive cues that remind them of their stigmatized group identity—may explain our findings^69–72^. Future experimental research, and, for example testing the attenuating effects of self-affirmation interventions^82^ on the relations we found, is needed to better understand the extent to which stereotype threat can explain our findings.

As our data are observational, we cannot exclude the possibility that items with and without low-SES relevant content differed on relevant, unobserved features. Although we controlled for key features known to affect low-SES students’ performance, there may have been unknown features that influenced our results. In addition, it is possible that items with money content require a different skill set than other items. In an experimental setting, it would be possible to test the effects of ecologically relevant content on math performance more systematically, by manipulating the content of items.

Our study may underestimate the extent to which ecologically relevant content actually impedes low-SES students’ math ability. When determining our plan for analyses, we decided to compute a student’s average test score as the matching criterion (or ‘true ability’) over items with all types of content^83^. This approach is recommended, because excluding items that are subject to the DIF-analyses from the matching criterion has been shown to impact the accuracy of DIF detection, increasing the risk for type I error^84^. However, this average across all items inevitable encompasses the biasing effect from items with ecologically relevant content—that is, those items that are associated with lower performance in our study. Since the results of our analyses have shown that ecologically relevant content disadvantages low-SES students’ math performance, it would be defensible to compute math ability using only those items *that do not have* ecologically relevant content. Following this procedure, the estimation of low-SES students’ true math ability would be higher than the estimation in our analyses, and the performance gap with items *that do have* ecologically relevant content would likely be larger.

Future research may explore the attentional, cognitive, and affective mechanisms explaining our findings. First, we need experimental research to understand the mixed evidence on the effects of ecologically relevant content on low-SES students’ test performance. A particularly instructive direction would be research examining the conditions in which ecologically relevant content enhances working memory, updating and cognitive flexibility, and how these abilities are related to other key processes involved in solving mathematical problems, focusing on transference from informal to formal knowledge and skills, responses to salience of content, and attention capture effects.

Next, it is important to investigate which content, in addition to money, food, and social interaction, may be biased against certain SES-groups, and which content is ‘neutral’ for all. In addition, future research should investigate whether our findings generalize to testing in other educational domains, such as science or language. More insight in hidden bias in educational tests promotes equal opportunities for students from all socioeconomic backgrounds.

In the future, it will also be important to investigate if the patterns we found are the same within all countries. Currently, we have focused on universal patterns because of the similarities in standardized tests and manifestations of SES across countries. However, there may be differences within countries in the effects we find, for example due to specific policies or practices. This is also particularly important when it comes to practical implications of our findings, such as adjusting policies related to testing.

Our results add to evidence that standardized test results can be biased by influences that are unrelated to students’ learning ability. Obviously, more knowledge and understanding to prevent bias in school tests is essential in promoting equality in education. However, these results also contribute to the societal debate of whether schools should continue to focus on comparing student performance on a specific test in absolute terms, or rather focus on the progress of each individual student. The unfair consequences of biased test results could also be reduced when all developmental trajectories are treated as equally valid as long as students are learning and continuing to progress, as proposed recently by Van Atteveldt^85^.

Our study provides an important contribution to investigating sources of social inequality in education by showing that content in math items related to money, food, and social interaction may contribute to unintended biases in math tests for students from low-SES backgrounds. This raises the question of whether items with this content should be avoided in math tests. Because it is common practice in international monitoring studies which track student development over the years, to include items with money, food, and social interaction content (e.g., TIMSS), simply excluding items with this type of content from tests is neither desirable nor feasible. In addition, since equipping students with critical life skills is an important goal of elementary education worldwide, conceptual and procedural understanding of money is a crucial part of what students need to learn. Consequently, when the goal of a math test is to assess the ability to engage in monetary transactions, omitting items with money is not feasible as well. Therefore, it is important to design interventions that could reduce or remove the bias of this content. We see two possible levels for interventions: a) teacher level (e.g., giving teachers tools to provide additional guidance to students during test taking) and b) test level (e.g., removal of specific content that may be particularly triggering, or adding specific instructions when using content that can produce bias). Developing and applying interventions that help to reduce bias in math tests could be a promising avenue to make math tests fairer and enhance social equality in education.

## Methods

### Preregistration

In line with current recommendations about research practices^86^, we preregistered the data source, definitions, and statistical plan for Differential Item Functioning (DIF) analyses of our study at the Open Science Framework (see https://osf.io/9eqkp/). This preregistration is less detailed than is common today. When we wrote this preregistration in 2018, we had less experience with this practice than we do now, and fewer templates and examples were available. In what follows, we highlight aspects of our research that could have been clearer in our preregistration, as well as deviations from our preregistration. In all analyses (initial preregistered analyses, main analyses on student-level and on item-level), we applied the data source, definitions and plan for DIF-analyses from this preregistration. In our initial preregistered analyses, we conducted DIF-analyses as preregistered. In addition, in our main analyses, we conducted analyses that were not preregistered (mixed logistic regression analyses and linear regression analyses, Tables 1–3; Figs. 1, 2).

### Participants

We used the data from Trends in International Mathematics and Science Studies (TIMSS). We used released items from cohort 2007 and 2011 (*N* = 5501,165) from all participating countries (57 in 2007, 58 in 2011). TIMSS defines its international target populations in terms of the amount of years of schooling students have received. The international target populations for TIMSS are 1) students in their fourth year of formal schooling, and 2) students in their eighth year of formal schooling. Because we had no specific hypothesis about years of formal schooling or the age when SES-background may bias math test outcomes, we included students from both of the available grades: grades 4 (average age 9.5 years), and 8 (average age 13.5 years). The ethics committee of the International Association for the Evaluation of Educational Achievement (IEA) has provided the study approval. In each country participating in TIMSS, the study protocol must also be approved by at least one national educational authority. In most countries, this national approval of the study protocol occurs in collaboration with ministries of education. During recruitment and planning contacts with schools, field staff inquired about school requirements for informing parents about their child’s participation in TIMSS. These requirements were categorized into three main approaches: Firstly, some schools opted for a notification method, simply sending parents a notification regarding their child’s participation. Secondly, there was a passive consent approach, where schools were mandated to request permission from parents for the child’s participation, with consent assumed unless a formal objection was raised. Lastly, there was an active consent approach, where schools were required to obtain formal parental consent before the child could take part in the assessment. However, the vast majority of schools chose to notify parents through a notification procedure.

### SES

Initially, our plan was to apply two proxies for SES, as recommended by the APA task force Socioeconomic Status^26^. Finding comparable indicators for socioeconomic background in international educational studies is difficult, for example, because socioeconomic background is defined differently across countries, and students may not know details about their parents’ educational level, occupational status, and income^87^. However, given the firm relation between socioeconomic background and academic achievement, reliable and valid indicators of socioeconomic status (SES) are essential for educational research^16^. One such indicator that is frequently used and recommended in cross-national educational research is the number of books at home^17–20^. For example, Heppt et al.^16^ showed that number of books at home is moderately correlated with income and occupational status (*r* = 0.35, Cohen’s *d* = 0.75), and parental educational level (*r* = 0.30, Cohen’s *d* = 0.63). These are modest correlations, indicating that this measure is not a perfect predictor of SES. One reason could be that cultural resources plays a relatively large role in the number of books present in a household compared to other indicators of SES. Wiberg et al.^88^ also point out that when one has access to SES information from official records, it is advisable to use it, and preferable to using the book measure. At the same time, however, it has already been regularly shown that this book measure is modestly correlated with SES (not only with cultural capital) and that for pragmatic reasons it is often the only possible measure, as is also the case in the datasets we use. Therefore, we applied this measure as our indicator for SES. Participants were asked to give an estimation of the number of books in their home (“About how many books are there in your home? Do not count magazines, newspapers, or your school books.”). Participants indicated their estimation by choosing one out of five categories: 0–10 books; 11–25 books; 26–100 books; 101–200 books, and more than 200 books. A higher score indicates a higher SES on a scale from 1–5 (low–high).

In addition, we planned to include a measure of parental educational level, based on students’ ratings of the educational level of their mother and father on the international ISCED-classification on a 6-point scale (1 = no education, 6 = university degree). However, before conducting any hypothesis tests, while analyzing descriptive statistics, we noticed that 20% to 25% of the participants indicated not knowing their parents’ educational level. Moreover, we suspected this variable to show selectivity in the missing values, with students in the lowest SES group having the most missing values (SES 1 on a scale of 1 to 5 based on the indicator number of books at home). A post-hoc analysis, suggested by reviewers, confirmed this expectation: more than 50 percent of students in the lowest SES group did not indicate their parents’ level of education. As our hypotheses specifically concern the performance of students living in low-SES conditions, we decided not to pursue parental education level as a proxy for SES. Thus prior to conducting hypothesis tests, we chose to use the number of books in home as our only indicator of socioeconomic status. A limitation of this measure is that unsystematic errors in estimates of number of books may be slightly larger in countries that are less wealthy^89^. Nonetheless, this measure is generally considered a reliable indicator for SES in cross-national studies^16,19,88^. Cross-national studies have shown that number of books in home is a consistent and robust proxy for socioeconomic background, related to resources available for education, home literacy, and academic support in families^18,90^.

### Classification of items

We define items with ‘low-SES ecologically relevant content’ as items with mathematical problems involving 1) money, 2) food, or 3) social interaction (e.g., competition, working together). We define items with ‘low-SES neutral content and problems’ as items with mathematical problems involving 1) word problems with neutral content (e.g., buttons, frogs) or 2) mathematical notation (e.g., 5631 + 286 = …). First, one researcher coded items according to these definitions. Second, a researcher who was not involved in this study rated these items on the same categories. The overlap between the first researcher’s rating and the second researcher’s rating was 82%, which is higher than the commonly recommended 80% agreement as the minimum acceptable interrater agreement^91^. The conflicts of judgments were evaluated until full agreement was reached.

### Linguistic features of items

In line with Haag et al.^10^, we coded all items concerning linguistic features on several levels. Regarding descriptive features, we counted for each item total words, number of different words, total number of characters, number of characters without spaces, average syllables per word, number of sentences, and average sentence length in words, applying an online tool provided by Textalyser (http://textalyser.net/). In addition, we coded all items on the use of academic words (1 = at least one academic word, 0 = no academic words), applying the Academic Word List^92^.

Furthermore, two general areas of mathematical language are frequently distinguished in literature. First, quantitative language (such as ‘many’, ‘fewer’, ‘less’, and ‘more’) is related to comparisons between groups and numbers^93^. Second, spatial language (such as ‘near’, ‘above’, and ‘before’), refers to relations between objects and numbers on a line^94^. We coded all items with regard to the use of quantitative language (1 = at least once, 0 = no), and spatial language (1 = at least once, 0 = no).

### Analyses on student-level

In our main analyses on student-level, We conducted mixed logistic regressions analyses with performance on an item (1 = correct answer, 0 = incorrect answer) as a dependent variable, students SES-background (scale 1–5; 1 = low, 5 = high) with relevance category (low-SES relevant vs. low-SES neutral) as an interaction term, students SES-background (scale 1–5; 1 = low, 5 = high) and average individual test score as between subjects factors, relevance category (low-SES relevant vs. low-SES neutral) as a within subjects factor, and features of items (word problem, item type, context domain, cognitive domain, total word count, number of different words, total number of characters, number of characters without spaces, average syllables per word, sentence count, average sentence length, academic words, quantitative language, spatial language, and country-dummies) as covariates. We conducted these analyses separately for grades 4 and 8.

### Analyses on item-level

To detect on an item level whether low-SES students are more likely to respond correctly when items contain low-SES ecologically relevant content giving their true math ability, we conducted Differential Item Functioning (DIF) analyses for SES-background. Items only show DIF if students from different backgrounds with the overall performance on the test have a different probability of giving the correct response on this specific question. We used the percentage of released items correctly answered on the math test as overall test score. This overall total test score has the advantage that the test scores for which the DIF is calculated have the same measurement error as the matching criterion^95^. We analyzed for each item separately whether there was DIF for SES-background, and if so, whether the DIF was in favor of the low-SES students or the high-SES students. We conducted DIF analyses with Mantel-Haenszel (MH) procedure. A statistically significant chi-square identifies DIF, resulting from comparing item performance in the low-SES groups with the high-SES group after matching on the total score. In addition, because applying more than one analysis to detect DIF is recommended in order to reduce the risk of Type I error, we decided to use Logistic Regression analyses (LR) as an additional method to detect DIF^96,97^. To detect uniform DIF, we applied LR with item response as dependent variable, and the total test score, and SES-background as independent variables^83^. We conducted DIF-analyses for all 161 items (Grade 4 and Grade 8, 2007 and 2011) using MH. By selecting students with the highest SES (SES = 5) and the lowest SES (SES = 1), we created a dichotomous measure for SES (1 = low, 0 = high), because MH does not allow scale variables. This procedure resulted in information for all 161 items about the occurrence of DIF to the disadvantage of low-SES (1 = yes, 0 = no), and odds (measure for the amount and direction of DIF) and allowed us to conduct analyses at the level of items. To control for important features of items that can affect low-SES students’ performance, we conducted linear regression analysis with DIF-odds as dependent variable, low-SES relevance as predictor, and all relevant variables (bold) in Table 3 as covariates.

### Reporting summary

Further information on research design is available in the Nature Research Reporting Summary linked to this article.

### Supplementary information

Supplementary file

Reporting summary

## Data Availability

This project utilized data from Trends in International Mathematics and Science Studies (TIMSS), collected by the International Association for the Evaluation of Educational Achievement (IEA). Data and documentation files of completed IEA studies are available at https://www.iea.nl/data. The data that support the findings of this study are openly available in the TIMSS data repository at https://timssandpirls.bc.edu/databases-landing.html^98^.

## Code availability

Definitions and statistical plan for Differential Item Functioning (DIF) analyses of our study can be accessed at the Open Science Framework (see https://osf.io/9eqkp/). All code used in the current study and in initial preregistered studies (SPSS) can be accessed at https://osf.io/yj3w7/?view_only=fa880317750341c2b1ab52d8ca42c094.
