# Supplementary material for: Math items about real-world content lower test-scores of students from families with low socioeconomic status
Source: NPJ Sci Learn. 2024 Mar 15;9:19. doi: 10.1038/s41539-024-00228-8 (PMC10943209; doi:10.1038/s41539-024-00228-8)
Supplement: Supplementary file 1 — Supplementary file [file 41539_2024_228_MOESM1_ESM.pdf]

Supplementary file

As preregistered, we conducted Differential Item Functioning (DIF) analyses for SES in the initial preregistered analyses in order to detect if ecologically relevant content biases test results on item-level. Initially we hypothesized that low-SES students might perform better on math test items with ecologically relevant content compared with their average performance on all math items. This is reflected in the direction of the research question in the preregistration:

"Do low-SES children perform better at math test items with low-SES relevant content and problems than expected based on their average performance on math?"

First, we conducted DIF-analyses with 20 randomly selected items containing low-SES ecologically relevant content (i.e., money, food, and social interaction), using similar procedures and definitions as described in the methods section. The only difference is that we used Plausible Values as matching criterium. we needed a measure for average math performance as matching criterion. TIMSS distinguishes between three cognitive domains—Knowing, Applying, and Reasoning—which represent different levels of math development and independently predict future math development, Knowing refers to basic concepts and procedures students need to know, while Applying refers to the ability to apply this basic knowledge in solving mathematical problems. Reasoning goes beyond solving routine problems and refers to the ability to solve complex problems that require multistep solutions[1]. For each domain, TIMSS estimates students' ability by estimating their Plausible Value. In the initial preregistered analyses (supplemental material), we considered these Plausible Values as a matching criterion for the DIF analyses[1-4], applying for each item the Plausible Value related to the item's cognitive domain. We applied MH and LR for each item, and only when both analyses for one item detected significant DIF, we concluded that the item showed DIF. In contrast to preregistered predictions, results showed that nine out of the 20 selected items with low-SES ecologically relevant content showed DIF to the disadvantage of low-SES students (45%) (see Supplementary table 1).

Supplementary table 1

Results from DIF-Analyses on items with low-SES ecologically relevant content.

Items			Results			
Number	Category	Content	LR Exp(B)	LR 95% CI	MH	DIF
1	Resources	Money, fair	0.670**	[0.543,0.803]	Flag A	DIF, disadvantage for low-SES
2	Resources	Market, shop	0.885**	[0.778,0.991]	Flag A	DIF, disadvantage for low-SES
3	Resources	Prize for a coat	0.836**	[0.757,0.915]	Flag A	DIF, disadvantage for low-SES
4	Resources	Prize for pencils	0.710**	[0.607,0.812]	Flag A	DIF, disadvantage for low-SES
5	Resources	Candies	0.775**	[0.704,0.846]	Flag A	DIF, disadvantage for low-SES
6	Resources	Money, lunch	1.022	[0.907,1.137]	.	
7	Resources	Money, rent	0.956	[0.818,1.094]	.	
8	Resources	Cake	1.076	[0.942,1.21]	.	
9	Resources	Costs of a trip	0.963	[0.848,1.077]	.	
10	Resources	Money	1.015	[0.888,1.141]	.	
11	Social interaction	Lottery	0.659**	[0.574,0.743]	Flag A	DIF, disadvantage for low-SES
12	Social interaction	Long jump competition	0.817**	[0.743,0.89]	Flag A	DIF, disadvantage for low-SES
13	Social interaction	Elections for school president	0.581**	[0.525,0.637]	Flag A	DIF, disadvantage for low-SES
14	Social interaction	Taking care of elderly	0.710**	[0.607,0.812]	Flag A	DIF, disadvantage for low-SES
15	Social interaction	Family	0.882*	[0.768,0.996]	.	
16	Social interaction	Running competition	0.968	[0.834,1.102]	.	
17	Social interaction	Game	0.995	[0.839,1.15]	.	
18	Social interaction	Trading with cards	1.062	[0.943,1.181]	.	
19	Social interaction	Sharing	0.935	[0.811,1.058]	.	
20	Social interaction	Basketball	1.039	[0.926,1.151]	.	

Note: Flag A = small levels of DIF. Items sorted by low-SES category and DIF.

LR showed that these effects within these nine items are large, indicating 12% (on the item showing least DIF) to 42% (on the item showing most DIF) lower chance for low-SES students to respond correctly on an item, compared to high-SES students with the same math ability. In addition, 11 items (55%) did not show DIF in relation to SES-background.

Next, because we expected no DIF for SES in items with content that is not ecologically relevant for low-SES students, we selected 20 items with ‘neutral’ content. We analyzed for each randomly selected neutral item whether there was DIF for SES-background, applying the same definitions and methodology as in our primary analyses. For the neutral items with mathematical notation, seven out of 10 items (70%) did not show DIF, two out of 10 items (20%) showed DIF to the disadvantage of low-SES students, and one item (10%) showed DIF in favor of low-SES students. For items with neutral items with word problems, eight items did not show DIF (80%), and two items (20%) showed DIF to the disadvantage of low-SES students (see Supplementary table 2). In sum, as expected, results did not suggest a pattern of DIF to the advantage or disadvantage of low-SES students in items with neutral content.

Supplementary table 2

Results from DIF-analyses on items with neutral content.

Items			Results			
Number	Content	Type of question	LR Exp(B)	LR 95% CI	MH	Decision
1	Pieces of cardboard	Word problem	0.620**	[0.546, 0.704]	Flag A	DIF, disadvantage for low-SES
2	Matches	Word problem	0.940	[0.849, 1.040]	.	
3	Boxes in a corner	Word problem	1.029	[0.913, 1.161]	.	
4	Temperature	Word problem	0.820*	[0.720, 0.933]	.	
5	Cat	Word problem	1.060	[0.917, 1.225]	.	
6	Ponds and frogs	Word problem	0.938	[0.849, 1.035]	.	
7	Tiles	Word problem	1.113	[0.994, 1.246]	.	
8	Cubes	Word problem	0.743**	[0.676, 0.817]	Flag A	DIF, disadvantage for low-SES
9	Length of a pipe	Word problem	0.781**	[0.703, 0.868]	.	
10	Staff members	Word problem	0.783**	[0.701, 0.874]	.	
11	Rotation	Math notation	0.846*	[0.754, 0.949]	.	
12	Fraction	Math notation	0.831**	[0.760, 0.909]	.	
13	Shape	Math notation	0.981	[0.891, 1.080]	.	
14	Number	Math notation	0.907	[0.804, 1.002]	.	
15	Fractions	Math notation	0.781	[0.686, 0.888]	.	
16	Fractions	Math notation	1.175*	[1.064, 1.296	Flag A	DIF in favor of low-SES
17	Figure	Math notation	0.865**	[0.785, 0.954]	Flag A	DIF, disadvantage for low-SES
18	Fractions	Math notation	0.725**	[0.658, 0.800]	Flag A	DIF, disadvantage for low-SES
19	Prime factors	Math notation	0.912	[0.830, 1.002]	.	
20	Fractions	Math notation	1.001	[0.882, 1.137]	.	

Note: Flag A = small levels of DIF. Items sorted by low-SES category and DIF.

In order to check the robustness of our unexpected findings regarding low-SES ecologically relevant items, we replicated this study by conducting the same primary analyses as in our preregistered research plan, with data from earlier waves of TIMSS (1999 and 2003 instead of 2007 and 2011). We used the same procedures and definitions as in our first analyses with these new datasets. Results showed the same pattern as in our first analyses: of 20 randomly selected items with

low-SES ecologically relevant content, eight showed DIF (40%) to the disadvantage of low-SES students see Supplementary table 3).

Supplementary table 3

Results from DIF-analyses on items with low-SES ecologically relevant content, replication with years 1999 and 2003.

Items			Results			
Number	Category	Content	LR Exp(B)	LR 95% CI	MH	Decision
1	Resources	Cake	0.801**	[0.708, 0.908]	Flag A	DIF, disadvantage for low-SES
2	Resources	Costs of magazines	0.494**	[0.447, 0.547]	Flag A	DIF, disadvantage for low-SES
3	Resources	Food	0.945	[0.891, 1.001]	Flag A	
4	Resources	Food	1.053	[0.931, 1.191]	.	
5	Resources	Selling apples	0.863	[0.868, 1.184]	.	
6	Resources	Cake	0.875	[0.761, 1.006]	.	
7	Resources	Ice-cream	0.830**	[0.730, 0.934]	.	
8	Resources	Trading	0.897	[0.787, 1.002]	.	
9	Resources	Money	0.962	[0.904, 1.023]	.	
10	Resources	Money	1.117	[0.972, 1.283]	.	
11	Social interaction	Trading	0.631**	[0.549, 0.725]	Flag A	DIF, disadvantage for low-SES
12	Social interaction	Comparison of persons' length	0.697**	[0.609, 0.797]	Flag A	DIF, disadvantage for low-SES
13	Social interaction	Game	0.691**	[0.551, 0.866]	Flag A	DIF, disadvantage for low-SES
14	Social interaction	Sharing sweets	0.698**	[0.579, 0.842]	Flag A	DIF, disadvantage for low-SES
15	Social interaction	Trading	0.556**	[0.525, 0.588]	Flag A	DIF, disadvantage for low-SES
16	Social interaction	Comparing paces	0.459**	[0.408, 0.517]	Flag A	DIF, disadvantage for low-SES
17	Social interaction	Sharing marbles	1.026	[0.921, 1.143]	.	
18	Social interaction	Sharing cherries	0.912	[0.821, 1.024]	.	
19	Social interaction	Game	1.006	[0.939, 1.079]	.	
20	Social interaction	Comparison between classes	0.870	[0.707, 1.070]	.	

Note. Flag A = small levels of DIF. Items sorted by low-SES category and DIF.

Supplementary Discussion

Results of our initial preregistered analyses, both from first analyses and a replication, indicate that items with low-SES ecologically relevant content do not enhance math-performance among low-SES students, but on the contrary, that these items seem to diminish their performance. In addition, results suggest that items with neutral context less often bias low-SES students' performance and if they do so, such bias is not consistently in the same direction: that is, in some cases it is positive, in others negative. Results of these initial preregistered analyses motivated further analyses.

Supplementary References

1. Olson, J. F., Martin, M. O., & Mullis, I. V. S. (eds.) *TIMSS technical report 2007* (TIMSS & PIRLS International Study Center, Boston College, 2008).
2. Chen, Y. F. & Jiao, H. Exploring the utility of background and cognitive variables in explaining latent differential item functioning: An example of the PISA 2009 reading assessment. *Educ. Assess.* **19**, 77–96 (2014).
3. Hauger, J. B. & Sireci, S. G. Detecting differential item functioning across examinees tested in their dominant language and examinees tested in a second language. *Int. J. Test.* **8**, 237–250 (2008).
4. Zhu, X. S., Rupp, A. A. & Gao, J. Differential item functioning analyses in large-scale educational surveys: Key concepts and modeling approaches for secondary analysts. *J. Res. Educ. Sci.* **56**, 91–127 (2011).
