# Supplementary material for: Math items about real-world content lower test-scores of students from families with low socioeconomic status
Source: NPJ Sci Learn. 2024 Mar 15;9:19. doi: 10.1038/s41539-024-00228-8 (PMC10943209; doi:10.1038/s41539-024-00228-8)
Supplement: Supplementary file 2 — Reporting summary [file 41539_2024_228_MOESM2_ESM.pdf]

nature portfolio

Corresponding author(s): Marjolein Muskens

Last updated by author(s): May 12, 2023

Reporting Summary

Nature Portfolio wishes to improve the reproducibility of the work that we publish. This form provides structure for consistency and transparency in reporting. For further information on Nature Portfolio policies, see our Editorial Policies and the Editorial Policy Checklist.

Statistics

For all statistical analyses, confirm that the following items are present in the figure legend, table legend, main text, or Methods section.

n/a	Confirmed	
☐	☒	The exact sample size (n) for each experimental group/condition, given as a discrete number and unit of measurement
☐	☒	A statement on whether measurements were taken from distinct samples or whether the same sample was measured repeatedly
☐	☒	The statistical test(s) used AND whether they are one- or two-sided<br>*Only common tests should be described solely by name; describe more complex techniques in the Methods section.*
☐	☒	A description of all covariates tested
☐	☒	A description of any assumptions or corrections, such as tests of normality and adjustment for multiple comparisons
☐	☒	A full description of the statistical parameters including central tendency (e.g. means) or other basic estimates (e.g. regression coefficient) AND variation (e.g. standard deviation) or associated estimates of uncertainty (e.g. confidence intervals)
☐	☒	For null hypothesis testing, the test statistic (e.g. F, t, r) with confidence intervals, effect sizes, degrees of freedom and P value noted<br>*Give P values as exact values whenever suitable.*
☒	☐	For Bayesian analysis, information on the choice of priors and Markov chain Monte Carlo settings
☒	☐	For hierarchical and complex designs, identification of the appropriate level for tests and full reporting of outcomes
☐	☒	Estimates of effect sizes (e.g. Cohen's d, Pearson's r), indicating how they were calculated

Our web collection on statistics for biologists contains articles on many of the points above.

Software and code

Policy information about availability of computer code

Data collection	Data from TIMSS 1999, 2003, 2007 and 2011 https://timssandpirls.bc.edu/timss-landing.html
Data analysis	SPSS 19

For manuscripts utilizing custom algorithms or software that are central to the research but not yet described in published literature, software must be made available to editors and reviewers. We strongly encourage code deposition in a community repository (e.g. GitHub). See the Nature Portfolio guidelines for submitting code & software for further information.

Data

Policy information about availability of data

All manuscripts must include a data availability statement. This statement should provide the following information, where applicable:

- Accession codes, unique identifiers, or web links for publicly available datasets
- A description of any restrictions on data availability
- For clinical datasets or third party data, please ensure that the statement adheres to our policy

This project utilized data from Trends in International Mathematics and Science Studies (TIMSS), collected by the International Association for the Evaluation of Educational Achievement (IEA). Data and documentation files of completed IEA studies are available at https://www.iea.nl/data. We preregistered the data source, definitions, statistical plan for Differential Item Functioning (DIF) analyses of our study at the Open Science Framework (see https://osf.io/9eqkp/).

Research involving human participants, their data, or biological material

Policy information about studies with human participants or human data. See also policy information about sex, gender (identity/presentation), and sexual orientation and race, ethnicity and racism.

Reporting on sex and gender	We did not report on sex and/or gender in this study.
Reporting on race, ethnicity, or other socially relevant groupings	We report on students' socioeconomic status (SES). Initially, our plan was to apply two proxies for SES, as recommended by the APA task force Socioeconomic Status (2007). Finding comparable indicators for socioeconomic background in international educational studies is difficult, for example, because socioeconomic background is defined differently across countries, and students may not know details about their parents' educational level, occupational status, and income (e.g., Torney-Purta et al., 2001). However, given the firm relation between socioeconomic background and academic achievement, reliable and valid indicators of socioeconomic status (SES) are essential for educational research (Heppt et al., 2022). One such indicator that is frequently used and recommended in cross-national educational research is the number of books at home (e.g., Brunello et al., 2017; Eriksson et al., 2021; Evans et al., 2010; Jerrim et al., 2014). Therefore, we planned to apply this measure as proxy for SES. Number of books captures access to cultural capital, an aspect of SES associated with educational achievement (Yang & Gustafsson, 2004). Participants were asked to give an estimation of the number of books in their home (“About how many books are there in your home? Do not count magazines, newspapers, or your school books.”). Participants indicated their estimation by choosing one out of five categories: 0–10 books; 11–25 books; 26–100 books; 101–200 books, and more than 200 books. A higher score indicates a higher SES on a scale from 1–5 (low–high).<br>In addition, we planned to include a measure of parental educational level, based on students' ratings of the educational level of their mother and father on the international ISCED-classification on a 6-point scale (1 = no education, 6 = university degree). However, before conducting any hypothesis tests, while analyzing descriptive statistics, we noticed that 20% to 25% of the participants indicated not knowing their parents' educational level. Moreover, we suspected this variable to show selectivity in the missing values, with students in the lowest SES group having the most missing values (SES 1 on a scale of 1 to 5). A post-hoc analysis, suggested by reviewers, confirmed this expectation: more than 50 percent of students in the lowest SES group did not indicate their parents' level of education. As our hypotheses specifically concern the performance of students living in low-SES conditions, we decided not to pursue parental education level as a proxy for SES. Thus prior to conducting hypothesis tests, we chose to use the number of books in home as our only indicator of socioeconomic status. One limitation of this measure is that unsystematic errors in estimates of number of books may be slightly larger in countries that are less wealthy (Eriksson et al., 2022). Nonetheless, this measure is generally considered a reliable indicator for SES in cross-national studies (Evans et al, 2020; Heppt et al., 2022; Wiberg et al., 2023). Cross-national studies have shown that number of books in home is a consistent and robust proxy for socioeconomic background, related to resources available for education, home literacy, and academic support in families (Beaton, 1996; Eriksson et al., 2021). We have not controlled for other socially relevant grouping.
Population characteristics	Participants of TIMSS are students in grade 4 and 8 from countries all over the world (e.g., participating countries in 2011: ustralia, Bahrain, Armenia, Botswana, Chile, Chinese Taipei, Finland, Georgia, Palestinian National Authority, Ghana, Honduras, Hong Kong, SAR, Hungary, Indonesia, Iran, Israel, Italy, Japan, Kazakhstan, Jordan, Korea Republic of, Lebanon, Lithuania, Malaysia, Morocco, Oman, New Zealand, Norway, Qatar, Romania, Russian Federation, Saudi Arabia, Singapore, Slovenia, South Africa, Sweden, Syria, Thailand, United Arab Emirates, Tunisia, Turkey, Ukraine, Macedonia, United States, England, United Arab Emirates (Dubai), United Arab Emirates (Abu Dhabi), Canada (Ontario), Canada (Quebec), Canada (Alberta).
Recruitment	IEA has collected the data. TIMSS assessments use national curricula as the major organizing concept. Assessments are based on comprehensive frameworks that are built in collaboration with participating countries to describe the knowledge and skills expected of students at fourth and eighth grades. National Research Coordinators play an important role in helping to develop the assessment questions and questionnaires, administering the assessment, reporting the results, and interpreting the findings within their own national context. These coordinators communicate with schools within their country. Participation is voluntary for countries, and for schools within these countries. Also, at participating schools, parents can indicate if they do not want their child to participate.
Ethics oversight	The organization responsible for the study protocol and ethics of TIMSS is the IEA (International Association for the Evaluation of Educational Achievement). The IEA is an international partnership of national research institutions, government agencies, scientists and analysts working to research, understand and improve education worldwide. Study approval and data protection. In each country participating in TIMSS, the study protocol must be approved by one or more educational authorities. These authorities also ensure that the investigation includes strict data protection procedures. In most countries, the approval of the study protocol occurs in collaboration with the ministries of education

Note that full information on the approval of the study protocol must also be provided in the manuscript.

Field-specific reporting

Please select the one below that is the best fit for your research. If you are not sure, read the appropriate sections before making your selection.

☐ Life sciences ☒ Behavioural & social sciences ☐ Ecological, evolutionary & environmental sciences

For a reference copy of the document with all sections, see nature.com/documents/nr-reporting-summary-flat.pdf

Behavioural & social sciences study design

All studies must disclose on these points even when the disclosure is negative.

Study description	Quantitative cross-sectional study. Full documentation of the many technical activities required to conduct TIMSS is provided in yearly technical Report reports at https://www.iea.nl/studies/iea/timss. Within this section, we cite information retrieved from these technical reports.
Research sample	We used the data from Trends in International Mathematics and Science Studies (TIMSS). We used released items from cohort 2007 and 2011 (N = 5,501,165) from all participating countries (57 in 2007, 58 in 2011). TIMSS defines its international target populations in terms of the amount of years of schooling students have received. The international target populations for TIMSS are 1) students in their fourth year of formal schooling, and 2) students in their eighth year of formal schooling. Because we had no specific hypothesis about years of formal schooling or the age when SES-background may bias math test outcomes, we included students from both of the available grades: grades 4 (average age 9.5 years), and 8 (average age 13.5 years).
Sampling strategy	In 2011, the following countries participated in TIMSS: Australia, Bahrain, Armenia, Botswana, Chile, Chinese Taipei, Finland, Georgia, Palestinian National Authority, Ghana, Honduras, Hong Kong, SAR, Hungary, Indonesia, Iran, Israel, Italy, Japan, Kazakhstan, Jordan, Korea Republic of, Lebanon, Lithuania, Malaysia, Morocco, Oman, New Zealand, Norway, Qatar, Romania, Russian Federation, Saudi Arabia, Singapore, Slovenia, South Africa, Sweden, Syria, Thailand, United Arab Emirates, Tunisia, Turkey, Ukraine, Macedonia, United States, England, United Arab Emirates (Dubai), United Arab Emirates (Abu Dhabi), Canada (Ontario), Canada (Quebec), Canada (Alberta).<br>National Research Coordinators play an important role in helping to develop the assessment questions and questionnaires, administering the assessment, reporting the results, and interpreting the findings within their own national context. These coordinators communicate with schools within their country. Participation is voluntary for schools. Also, at participating schools, parents can indicate if they do not want their child to participate.<br>Each country participating in TIMSS needs a plan for defining its national target population and applying the TIMSS sampling methods to achieve a nationally representative sample of schools and students. The development and implementation of the national sampling plan is a collaborative exercise involving the country's National Research Coordinator (NRC) and TIMSS sampling experts. For most countries, TIMSS requirements are met with a school sample of 150 schools and a student sample of 4,000 students for each target grade. Depending on the average class size in the country, one class from each sampled school may be sufficient to achieve the desired student sample size.
Data collection	In 1999, 2003, 2007 and 2011, TIMSS was conducted with pen and paper. Research assistants helped classroom teachers administer the tests. The test items were developed by the IEA in collaboration with national research coordinators.
Timing	Each year (1999, 2003, 2007, and 2011), the data collection took place in the first quarter of a year, starting in January
Data exclusions	School-Level Exclusions. Although it is expected that very few schools will be excluded from the national target population, NRCs are permitted to exclude schools on the following grounds when they consider it necessary:<br>• Inaccessibility due to their geographically remote location<br>• Extremely small size (e.g., four or fewer students in the target grade)<br>• Offering a grade structure, or curriculum, radically different from the mainstream educational system<br>• Providing instruction solely to students in the student-level exclusion categories listed below (e.g., catering only to special needs students)<br>Student-Level Exclusions. The international within-school exclusion rules are specified as follows:<br>• Students with functional disabilities — These are students who have physical disabilities such that<br>they cannot perform in the TIMSS testing situation. Students with functional disabilities who are able to perform should be included in the testing.<br>• Students with intellectual disabilities — These are students who are considered, in the professional opinion of the school principal or by other qualified staff members, to have intellectual disabilities or who have been tested as such. This includes students who are emotionally or mentally unable to follow even the general instructions of the test. Students should<br>not be excluded solely because of poor academic performance or normal disciplinary problems.<br>It should be noted that students with dyslexia, or other such learning disabilities, should be accommodated in the test situation if possible, rather than excluded.<br>• Non-native language speakers — These are students who are unable to read or speak the language(s) of the test and would be unable to overcome the language barrier in the test situation. Typically, a student who has received less than one year of instruction in the language(s) of the test should be excluded.
Non-participation	To minimize the potential for non-response bias, TIMSS aims for 100 percent participation by sampled schools, classrooms, and students, while recognizing that some degree of non-participation may be unavoidable. For a national sample to be fully acceptable it must have either:<br>• A minimum school participation rate of 85 percent, based on originally sampled schools AND<br>• A minimum classroom participation rate of 95 percent, from originally sampled schools and replacement schools AND<br>• A minimum student participation rate of 85 percent, from sampled schools and replacement schools<br>OR<br>• A minimum combined school, classroom, and student participation rate of 75 percent, based on originally sampled schools (although classroom and student participation rates may include replacement schools)<br>Classrooms with less than 50 percent student participation are deemed to be not participating.

Technical report show that it occurs very occasionally that a country fails to have more that 85% of schools and is therefore excluded. Participation rates of students and classes are generally met.

Randomization	N.A.

Reporting for specific materials, systems and methods

We require information from authors about some types of materials, experimental systems and methods used in many studies. Here, indicate whether each material, system or method listed is relevant to your study. If you are not sure if a list item applies to your research, read the appropriate section before selecting a response.

Materials & experimental systems

n/a	Involved in the study
☒	☐ Antibodies
☒	☐ Eukaryotic cell lines
☒	☐ Palaeontology and archaeology
☒	☐ Animals and other organisms
☒	☐ Clinical data
☒	☐ Dual use research of concern
☒	☐ Plants

Methods

n/a	Involved in the study
☒	☐ ChIP-seq
☒	☐ Flow cytometry
☒	☐ MRI-based neuroimaging
